# James St. John Gage Parsons

**Published:** 1905-09

**Authors:** 


### JAMES St. JOHN GAGE PARSONS, M.D. Chicago, F.R.C.S.,

A RETIRED member of the profession, who qualified in 1843, was not much known to his colleagues of the present day. He did good work in connection with cholera in Bristol in 1849, and was held in high esteem by those who knew him. He died at his residence, Cotham Hill, at the advanced age of 87, having been born in Bristol in 1818. He was connected with the Bristol School of Anatomy and Medicine from 1837 to 1842, and was for a time House-Surgeon to the Bristol General Hospital. He was a cultured man, who took much interest in artistic and many scientific subjects: he was a good botanist and a keen theologian.

